# Identification of a Prenyl Chalcone as a Competitive Lipoxygenase Inhibitor: Screening, Biochemical Evaluation and Molecular Modeling Studies

**DOI:** 10.3390/molecules26082205

**Published:** 2021-04-12

**Authors:** Maria Luiza Zeraik, Ivani Pauli, Luiz A. Dutra, Raquel S. Cruz, Marilia Valli, Luana C. Paracatu, Carolina M. Q. G. de Faria, Valdecir F. Ximenes, Luis O. Regasini, Adriano D. Andricopulo, Vanderlan S. Bolzani

**Affiliations:** 1 Laboratório de Fitoquímica e Biomoléculas (LabFitoBio), Departamento de Química, Universidade Estadual de Londrina (UEL), Londrina, PR 86051-990, Brazil; 2 Núcleo de Bioensaios, Biossíntese e Ecofisiologia de Produtos Naturais (NuBBE), Departamento de Química Orgânica, Instituto de Química, Universidade Estadual Paulista (UNESP), Araraquara, SP 14800-060, Brazil; luizdutra_qf@yahoo.com.br (L.A.D.); raqsabara@hotmail.com (R.S.C.); mariliava@gmail.com (M.V.); luisregasini@gmail.com (L.O.R.); 3 Laboratório de Química Medicinal e Computacional (LQMC), Centro de Pesquisa e Inovação em Biodiversidade e Fármacos (CIBFar), Instituto de Física de São Carlos, Universidade de São Paulo (USP), São Carlos, SP 13563-120, Brazil; ivanipauli@gmail.com (I.P.); aandrico@ifsc.usp.br (A.D.A.); 4 Departamento de Química, Faculdade de Ciências, Universidade Estadual Paulista (UNESP), Bauru, SP 17020-360, Brazil; luanachiquetto@hotmail.com (L.C.P.); carolina.quinello@gmail.com (C.M.Q.G.d.F.); valdecir.ximenes@unesp.br (V.F.X.); 5 Instituto de Biociências, Letras e Ciências Exatas, Universidade Estadual Paulista (UNESP), São José do Rio Preto, SP 15054-000, Brazil

**Keywords:** inflammation, docking, NuBBE database, chalcones

## Abstract

Cyclooxygenase (COX) and lipoxygenase (LOX) are key targets for the development of new anti-inflammatory agents. LOX, which is involved in the biosynthesis of mediators in inflammation and allergic reactions, was selected for a biochemical screening campaign to identify LOX inhibitors by employing the main natural product library of Brazilian biodiversity. Two prenyl chalcones were identified as potent inhibitors of LOX-1 in the screening. The most active compound, (*E*)-2-*O*-farnesyl chalcone, decreased the rate of oxygen consumption to an extent similar to that of the positive control, nordihydroguaiaretic acid. Additionally, studies on the mechanism of the action indicated that (*E*)-2-*O*-farnesyl chalcone is a competitive LOX-1 inhibitor. Molecular modeling studies indicated the importance of the prenyl moieties for the binding of the inhibitors to the LOX binding site, which is related to their pharmacological properties.

## 1. Introduction

Lipoxygenases are a family of iron-containing dioxygenases which are widely distributed in both animal and plant species. Human 5-lipoxygenase (5-LOX) is involved in the metabolism of arachidonic acid to leukotrienes, which are potent mediators of inflammation and allergic reactions [1,2]. Plants also contain numerous lipoxygenases, with at least eight having been identified in soybean (*Glycine max*), such as soybean lipoxygenase-1 (LOX-1), all of which use linoleic acid as a substrate [3,4]. Two important approaches for the design and synthesis of anti-inflammatory agents are based on the inhibition of two enzymes, cyclooxygenase (COX) and lipoxygenase, both of which are involved in the metabolism of arachidonic acid. Cyclooxygenase (COX) catalyzes the conversion of arachidonic acid to prostaglandins, with COX-1 being the constitutive isoform that is expressed in most tissues, while COX-2 is only expressed when induced by the response to inflammation [5].

Selective inhibitors of the COX-2 isoform have been developed as anti-inflammatory drugs, such as celecoxib (Celebrex) and rofecoxib (Vioxx), and are characterized as nonsteroidal anti-inflammatory drugs (NSAIDs). However, their use is associated with an increased risk of ulcerations, heart attacks, and strokes [6,7]. The inhibition of lipoxygenase is an alternative for the treatment of inflammatory processes, and it can also be a combination strategy in conjunction with COX-2 inhibitors [8,9,10,11]. Zileuton (Zyflo^®^), which is available in the USA for the treatment of asthma, is currently the only 5-LOX inhibitor marketed for therapy in humans. Nevertheless, zileuton shows low potential in other diseases, such as allergic rhinitis, rheumatoid arthritis, and inflammatory bowel disease, revealing a great need for novel inhibitors of 5-LOX [12].

As has been extensively reviewed, biodiversity provides exclusive chemical scaffolds for drug discovery [13,14]. Chalcones are known for their various biological activities, including antioxidant, anticancer, antimicrobial, anti-inflammatory, and antiprotozoal activities [15,16,17,18,19,20]. Prenylated chalcones are found as natural products, such as the derricidin and isocordoin used in this work as an inspiration, and they and others could be retrieved from the natural product database of Brazilian biodiversity (Nuclei of Bioassays, Ecophysiology and Biosynthesis of Natural Products Database, NuBBE_DB_) [21,22]. In the present work, we report the screening for LOX-1 inhibitors and the identification of a chalcone scaffold with prenyl groups as competitive inhibitors. The use of a polarography assay was applied in order to further explore the anti-inflammatory potential of the most potent compounds. Together with the in vitro experiments, molecular modeling studies were also conducted in order to reveal the molecular features related to the binding of this inhibitor to the LOX binding site.

## 2. Results and Discussion

The present work is part of a screening campaign to identify biologically-active natural products from Brazilian biodiversity to be used as models for further optimization in a drug discovery pipeline. A combination of in vitro assays, enzyme kinetics, and molecular modeling studies were used to evaluate the molecular reasons for the LOX inhibition activity of the most promising compound.

For the in vitro screening to identify the inhibitors of the enzymatic activity of LOX-1, a spectrophotometric assay was employed in order to measure the conjugated diene formed by linoleic acid oxidation. Soybean LOX-1, which converts linoleic acid to 13-hydroperoxylinoleic acid, is inhibited by NSAIDs in a qualitatively similar way to that of the rat mast cell line 5-LOX, and it may be used as a reliable screening target for this activity [23]. The screening was performed with compounds from Brazilian biodiversity (NuBBE_DB_) [15,16] and synthetic derivatives previously synthesized by using natural products as models. In this context, a variety of natural products were evaluated, such as triterpenes, chalcones, casearins, alkaloids, guanidines, and flavonoids. Among all of the screened compounds, prenyl chalcones were identified as LOX-1 inhibitors.

Hydroxyl chalcones (**1**–**3**) were previously obtained by Claisen–Schmidt condensation [24] followed by prenylation to obtain chalcones **4**–**6**. Prenyl chalcones and their precursor hydroxyl chalcones were evaluated in order to verify the relevance of the prenyl group for LOX-1 inhibition. Prenyl units are frequently found in natural products, and are associated with an increase in hydrophobicity and facilitated interaction with cell membranes. LOX-1 inhibition by (*E*)-2-*O*-farnesyl chalcone (**6**, IC_50_ = 5.7 µM) was twofold more potent than 3-*O*-geranyl chalcone (**5**, IC_50_ = 11.8 µM), and >10-fold more potent than 2-*O*-prenyl chalcone (**4**, IC_50_ > 50.0 µM). Chalcones containing hydroxyl groups did not show significant inhibitory effects on LOX-1, regardless of the position of these groups (Table 1). Compound **6** was almost twofold more potent than the positive control, curcumin (IC_50_ = 10.1 µM), and twofold less potent than the positive control, nordihydroguaiaretic acid (NDGA) (IC_50_ = 2.7 µM). The graph demonstrating the inhibitory activity of the compounds on LOX-1 at different concentrations is available online in the Supplementary Materials (Figure S1).

The results indicate that a larger extension of the prenyl chains is directly related to an enhanced inhibition of LOX-1. The extension of this chain results in higher lipophilicity and an increase in the capacity to perform hydrophobic interactions in the binding site. The binding site of LOX shows that it is primarily composed of hydrophobic amino acids, as will be discussed further in this paper within the molecular docking evaluation, specifying its preference for hydrophobic ligands, such as **6** and **5**. Lipophilicity is an important physicochemical property which is often related to the bioactivity of the compounds. The tendency observed herein is similar to that previously reported for curcumin analog LOX inhibitors [25]. The log *P* values were calculated based on the consensus method provided by the SwissADME web tool [26,27]. The increase in the molecular hydrophobicity of prenyl chalcones (log *P*) was correlated with the more potent inhibitory activities of the compounds, as shown in Table 1. A higher hydrophobicity was also previously correlated with a more potent antileishmanial activity for these compounds [24].

**Figure 1:**
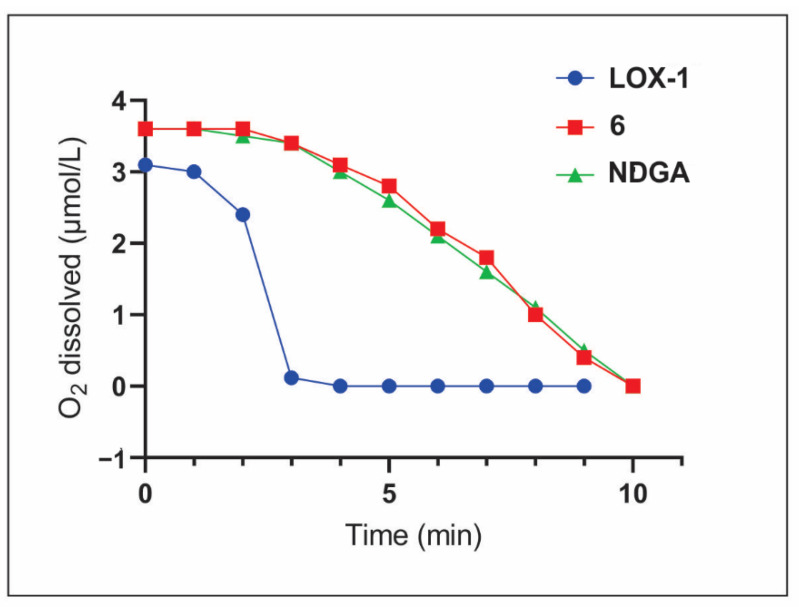
The activity of LOX-1 (1 µM) was monitored by O_2_ consumption. LOX-1 inhibition led to a lower rate of oxygen consumption, which was observed in the presence of (*E*)-2-*O*-farnesyl chalcone (**6**) and nordihydroguaiaretic acid (NDGA), both at 10 µM.

The catalytic activity of LOX-1 comprises the conversion of its substrate to polyunsaturated molecules by the consumption of oxygen atoms, which is a limiting rate of that mechanism. A polarography-based assay was used to evaluate the rate of the oxygen consumption during LOX-1 inhibition, during which oxygen consumption is associated with the formation of a hydroperoxyl group [28]. In order to evaluate the decrease in oxygen consumption upon LOX-1 inhibition by the prenyl chalcone **6**, an O_2_-selective electrode coupled to an oxygen monitoring probe was employed. A delay of the oxygen consumption was detected in the presence of prenyl chalcone **6** at 10 µM, to a similar extent to that of the positive control NDGA, which suggests a decline in LOX-1 catalysis and a decrease in the detectable polyunsaturated product (Figure 1).

Enzyme kinetics studies were carried out in order to determine the mechanism of inhibition of the most potent compound **6**. Primarily, the Michaelis–Menten constant (*K*_M_) and maximum velocity values (*V_max_*) were determined for the enzymatic activity without an inhibitor (*K*_M_ = 45.8 µM and *V*_max_ = 10 µmol/min). Increasing the concentrations of **6** (5–10 µM) allowed us to confirm the competitive mechanism against LOX-1 inhibition, with a *K*_i_ = 7 µM.

**Figure 2:**
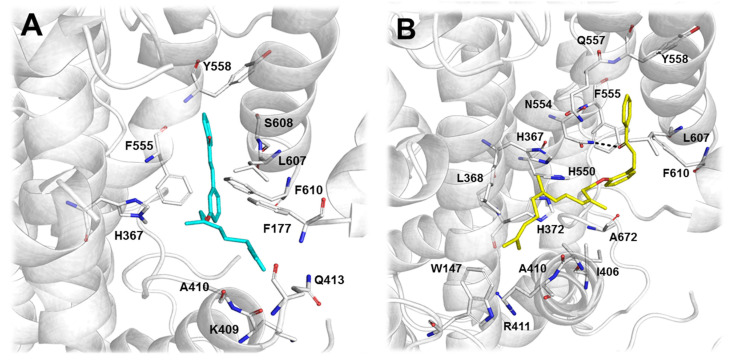
3D representation of the top-scored docking poses of **5** in cyan (**A**) and **6** in yellow (**B**), rendered as stick models in the human 5-LOX binding site (PDB ID: 3V99). The relevant binding site residues are highlighted and represented by the respective letter code: A (Ala) = alanine; F (Phe) = phenylalanine; H (His) = histidine; I (Ile) = isoleucine; K (Lys) = lysine; L (Leu) = leucine; N (Asn) = asparagine; Q (Gln) = glutamine; R (Arg) = arginine; S (Ser) = serine; Y (Tyr) = tyrosine; W (Trp) = tryptophan.

Molecular docking studies were performed in order to assess the molecular connections related to the experimental results obtained. In general, the binding site of eukaryotic LOXs is conservative, allowing us to use LOX-5 as structural model to determine ligand interactions [29,30]. The binding site of LOX-5 is primarily composed (approximately 50%) of hydrophobic amino acids (Trp147, Phe169, Phe177, Leu179, Phe229, Leu368, Ile406, Ala410, Phe555, Ala603, Leu607, Phe610, Val671, and Ala672), clarifying the increased potency for the hydrophobic compounds **6** and **5**. The increased potency of **6** compared to **5** is justified by the enhanced number of hydrophobic interactions due to the increased length of the prenyl chain, as shown in Figure 2. The docking simulations showed that **5** (Figure 2A), a chalcone with two prenyl groups at position 3 of ring B, interacts with the LOX binding cavity, where the chalcone moiety interacts with His367, Phe555, Tyr558, Leu607, and Phe610, while the prenyl chain is stabilized by the nonpolar residues Phe177, Ala410, Gln413 and Lys409. Compound **6** (Figure 2B) adopts a similar conformation to that of **5** in the LOX binding cavity. The same residues—His367, Phe555, Tyr558, Leu607, and Phe610—interact with the chalcone moiety; an additional hydrogen bond, formed between the chalcone carbonyl oxygen and the Asn554 side-chain nitrogen atom, contributes to the stabilization of the compound. Compound **6** presents a longer and more hydrophobic substituent in ring B at position 2, which is composed of three prenyl groups. Consequently, a higher number of protein-ligand hydrophobic interactions take place. In this case, the hydrophobic chain is stabilized by the nonpolar residues Trp147, Leu368, Ile406, Ala410, and Ala672, in addition to the basic amino acids Arg411, His372, and His550. The molecular docking study corroborated the previous experimental results by revealing an increased number of intermolecular interactions of **6** (ΔG_binding_ = −41.84) and the binding site compared to that of compound **5** (ΔG_binding_ = −38.15).

Previous studies performed computational approaches to screen chalcone and flavone derivatives for their potential as inhibitors of 5-LOX, and showed the lipoxygenase activity inhibition. This study was important to identify prenyl chalcones as inhibitors of LOX. The mode of binding for chalcones was assessed in order to verify the importance of the prenyl moieties for binding. The mode of binding was similar to other chalcones previously reported in the literature [31].

## 3. Materials and Methods

### 3.1. Synthesis of Compounds

Compound **1**–**3** and prenylchalcones **4**–**6** were synthesized as described previously [24]. The NMR data for compound **3** are shown below:

(*E*)-4-hydroxychalcone (16). Yellow solid, 33% yield. 1H NMR (500 MHz, DMSO-d6), δH 7.62 (d; J = 7.5 Hz, H-2/H-6), 6.82 (d; J = 8.5 Hz, H-3/H-5), 8.08 (dd; J = 1.5, 7.5 Hz, H-2′/H-6′), 7.53 (dd; J = 7.5, 7.5 Hz, H-3′/H-5′), 7.69 (m; H-4′/H-α/H-β). 13C NMR (500 MHz, DMSO-d6), δC 125.7 (C-1), 128.3 (C-2/C-6), 115.8 (C-3/C-5), 160.1 (C-4), 137.9 (C-1′), 130.9 (C-2′/C-6′), 128.6 (C-3′/C-5′), 132.7 (C-4′), 118.4 (C-α), 144.5 (C-β), 189.0 (C-β’).

### 3.2. LOX Bioassay

The ability of chalcones **1**–**6** to act as LOX-1 inhibitors was evaluated according to a method from Ha et al. (2004) [28]. Initially, 2.910 mL Tris-HCl buffer (0.1 mM (pH 8.0), Sigma-Aldrich, Brazil) was added to a quartz cuvette, followed by the addition of 25 μL LOX-1 (final concentration 1 μM, Sigma-Aldrich, Brazil) and 10 μL of the compounds tested (the final concentration ranged from 1 to 20 μM). The solution was incubated for 5 min at 25 °C. The reaction was started with the addition of linoleic acid (55 μL, 1 mM, Sigma-Aldrich, Brazil), and the LOX-1 activity was monitored at λ = 221 nm, pH 8.0, and 25 °C by an absorbance spectrophotometer in UV-Vis (Perkin Elmer Lambda 22, Waltham, MA, USA). For the determination of K_M_, varying substrate concentrations were used (linoleic acid, 13.7–91.7 µM) and 1 µM LOX-1. Nordihydroguaiaretic acid (NDGA, CAS Number: 500-38-9, Sigma-Aldrich, St. Louis, MO, USA ≥97%) was used as a positive control. The spectrophotometric measurements at λ = 221 nm are associated with the conjugated double bonds formed from the linoleic acid substrate.

### 3.3. Oxygen Consumption by the Catalytic Activity of LOX

The oxygen consumption was monitored using an O_2_-selective electrode coupled to a YSI 5300 Oxygen Monitor (Yellow Springs, OH, USA) at 25 °C. The reactions were initiated by adding O_2_ (100 μM), followed by the addition of LOX (20 nM) in 0.1 M Phosphate buffered saline (PBS), with pH 7.4 at 25 °C and the compounds being tested [32].

### 3.4. Docking Simulation

The 3D chemical structure of each compound was generated using the standard tools available in the molecular modeling software package Sybyl 8.0 [33]. The conformational energy was minimized using the Tripos force field and Powell’s method. The partial atomic charges were calculated using the Gasteiger–Hückel method, which is available in Sybyl 8.0, considering the molecules to be in an implicit aqueous environment (a dielectric constant of 80.0). The simulations were carried out using GOLD [34], a software package based on a genetic algorithm to explore the ligand conformational space. The protein structure of 5-LOX was PDB ID: 3V99, available at the Protein Data Bank (PDB). The protein was prepared for the docking studies by adding hydrogen atoms, removing the water, and cocrystallizing the inhibitors. The enzyme–inhibitor interactions within a radius of 12 Å centered on the PHE177 CZ atom were evaluated. The binding site was defined according to co-crystalized ligand arachidonic acid. The docking poses were obtained by applying the ChemScore fitness function. LIGPLOT [35] was used to identify the contacts among the inhibitors and LOX.

### 3.5. Theoretical Calculation of log P

The molecular hydrophobicity of the chalcone derivatives was calculated based on their log *P* values (partitioning coefficient in n-octanol/water) based on the consensus method (arithmetic mean of the values predicted by the following proposed methods: XLOGP3, WLOGP, MLOGP, SILICOS-IT, and iLOGP), and was performed using the SwissADME web tool [26,27].

## 4. Conclusions

This paper contributes to the field of inflammatory diseases with information on the molecular features which are most important for the development of LOX inhibitors. The LOX inhibition strategy is an important advancement to overcome the unwanted effects of anti-inflammatories that are selective for COX-2 inhibition. The research on LOX inhibitors published herein is important to address the treatment of inflammatory diseases for which the current drugs lack efficacy. The initial approach employing an in vitro screening was effective in identifying potent LOX-1 inhibitors, namely, (*E*)-2-*O*-farnesyl chalcone (**6**, IC_50_ = 5.7 µM) and 3-*O*-geranyl chalcone (**5**, IC_50_ = 11.8 µM). Next, the enzyme kinetics study allowed for the determination of the mode of action of the competitive inhibitor **6**, providing information on the binding site to be explored in the computational studies. The molecular reasons for the binding of compounds **5** and **6** to lipoxygenase were analyzed by computational molecular docking studies, highlighting the contribution of the prenyl moieties to the binding.

Finally, it is worthy to note that compound **6** was not more effective than NDGA, a well-established control for lipoxygenase inhibition. However, some advances can be expected, as chalcone derivatives showed lower cell toxicity [36]. On the other hand, the usefulness of NDGA is limited due to its toxicity. For example, its long-term use has been correlated with liver damage and kidney dysfunction [37].

## Figures and Tables

**Table 1:** IC_50_ values for the inhibition of lipoxygenase-1.

Compound	Structure	IC_50_ (µM) ^a^	Calculated log *P* ^b^
**1**	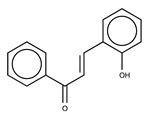	>50	3.09
**2**	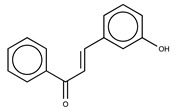	>50	2.87
**3**	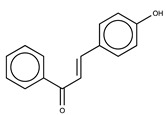	>50	2.87
**4**	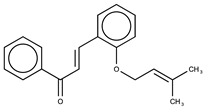	>50	4.52
**5**	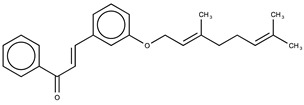	11.8 ± 0.9	5.94
**6**	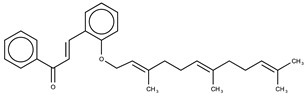	5.7 ± 0.8	7.46
**Curcumin**	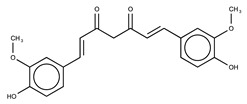	10.1 ± 0.5	
**Nordihydroguaiaretic acid (NDGA)**	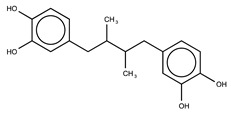	2.7 ± 0.5	

^a^ The values represent the mean and standard deviation of three independent experiments. ^b^ Calculated by the consensus method provided by the SwissADME web tool [26,27].

## Data Availability

Not applicable.

## Supplementary Materials

Supplementary Materials are available online.

## Sample Availability

Samples of the compounds are available from the authors.
