# Supplementary figures and images for: Identification of a Prenyl Chalcone as a Competitive Lipoxygenase Inhibitor: Screening, Biochemical Evaluation and Molecular Modeling Studies
Source: Molecules. 2021 Apr 12;26(8):2205. doi: 10.3390/molecules26082205 (PMC8069166; doi:10.3390/molecules26082205)

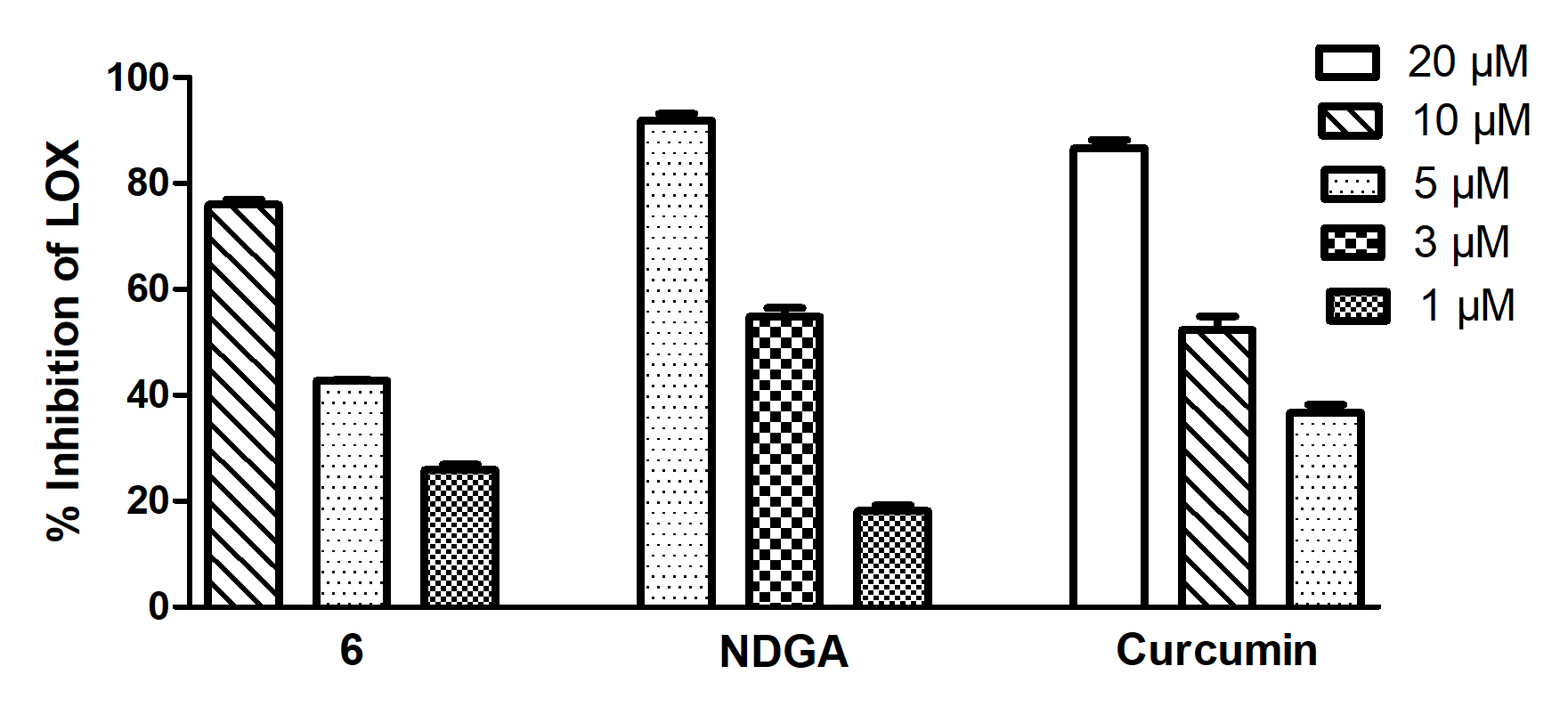

Supplement: Supplementary file 1 [file molecules-26-02205-s001.jpg]
